# *In vivo* Raman spectral analysis of impaired cervical remodeling in a mouse model of delayed parturition

**DOI:** 10.1038/s41598-017-07047-5

**Published:** 2017-07-28

**Authors:** Christine M. O’Brien, Jennifer L. Herington, Naoko Brown, Isaac J. Pence, Bibhash C. Paria, James C. Slaughter, Jeff Reese, Anita Mahadevan-Jansen

**Affiliations:** 1 Department of Biomedical Engineering, Vanderbilt University, Nashville, TN 37232 USA; 2 Biophotonics Center, Vanderbilt University, Nashville, TN 37232 USA; 3 Department of Pediatrics, Vanderbilt University Medical Center, Nashville, TN 37232 USA; 4 Department of Biostatistics, Vanderbilt University Medical Center, Nashville, TN 37232 USA; 5 Department of Cell and Developmental Biology, Vanderbilt University Medical Center, Nashville, TN 37232 USA

## Abstract

Monitoring cervical structure and composition during pregnancy has high potential for prediction of preterm birth (PTB), a problem affecting 15 million newborns annually. We use *in vivo* Raman spectroscopy, a label-free, light-based method that provides a molecular fingerprint to non-invasively investigate normal and impaired cervical remodeling. Prostaglandins stimulate uterine contractions and are clinically used for cervical ripening during pregnancy. Deletion of cyclooxygenase-1 (Cox-1), an enzyme involved in production of these prostaglandins, results in delayed parturition in mice. Contrary to expectation, Cox-1 null mice displayed normal uterine contractility; therefore, this study sought to determine whether cervical changes could explain the parturition differences in Cox-1 null mice and gestation-matched wild type (WT) controls. Raman spectral changes related to extracellular matrix proteins, lipids, and nucleic acids were tracked over pregnancy and found to be significantly delayed in Cox-1 null mice at term. A cervical basis for the parturition delay was confirmed by other *ex vivo* tests including decreased tissue distensibility, hydration, and elevated progesterone levels in the Cox-1 null mice at term. In conclusion, *in vivo* Raman spectroscopy non-invasively detected abnormal remodeling in the Cox-1 null mouse, and clearly demonstrated that the cervix plays a key role in their delayed parturition.

## Introduction

Pregnancy, labor, and the delivery of offspring (parturition) are highly regulated processes in all species. However, the mechanisms underlying the progression to term or preterm birth and onset of labor remain incompletely understood. Spontaneous preterm birth is considered a clinical syndrome that has multiple causes, including infection, cervical insufficiency, uterine overdistension, and others^1^. In all instances, cervical remodeling and dilation are required for successful expulsion of the fetus^2^. The cervix is endowed with important structural properties that ensure a tightly closed womb until the fetus is ready for delivery, and to act as a barrier against external infection. At the beginning of pregnancy, the cervix is a stiff, rigid cylinder with a highly cross-linked extracellular matrix that provides mechanical strength to maintain cervical closure^2, 3^. Over the course of pregnancy, this collagen-dense matrix is transformed into a compliant structure that allows passage of a fetus. Normal cervical remodeling during pregnancy involves a significant transition in mature to immature collagen cross-links^4^, a reduction in proteoglycans which control packing of collagen fibrils, a significant increase in the glycosaminoglycan hyaluronan^5^, and a significant increase in tissue hydration^6, 7^. Circulating as well as local hormone/endocrine levels are known to orchestrate these cervical changes^3, 8–11^, but a full understanding remains elusive.

Cyclooxygenase (Cox)-derived prostaglandins (PGs) serve as key signaling molecules during pregnancy^12–14^. PGF2α plays an important role in the involution of the ovarian corpus luteum (luteolysis) in mice and subsequent decline in progesterone signaling leading to uterine contractions^12, 15, 16^. However, there is conflicting evidence for PGs as mediators of cervical ripening. Topical prostaglandin E (PGE) application is a potent stimulus for the induction of cervical ripening in women and animal models^9, 17–20^, and PGs are required for cervical maturation during inflammation-induced preterm birth in mice^21^. Conversely, expression of Cox-1 and Cox-2 enzymes in the cervix is not increased at term in normal mouse models of pregnancy and neither are the concentrations of PGs or their receptors^21^. Furthermore, PG concentrations in cervical mucus do not increase prior to parturition in women^22–24^. In addition, seminal fluid has high levels of PGs but has not been shown to promote cervical ripening^25^. A greater understanding of PG-mediated changes in the cervix is needed to clearly understand normal remodeling physiology and reduce adverse pregnancy outcomes.

Classical research approaches for investigating physiology are too invasive to be used in pregnant women; therefore, animal models inform much of our current knowledge regarding hormone/endocrine factors in pregnancy and cervical maturation^26, 27^. Two cyclooxygenase isoforms, Cox-1 and Cox-2, are responsible for catalyzing arachidonic acid to form prostaglandin H2 (PGH2), which is promptly converted by various synthases into specific PG species. Cox-1 is generally considered a constitutively expressed enzyme, whereas Cox-2 is induced in response to inflammation and other stimuli but is otherwise not active during pregnancy. Mice lacking the gene encoding Cox-1 (*Ptgs1*) exhibit delayed parturition^12, 13, 28, 29^. Previous reports in mice suggest that the absence of prostaglandins normally produced by Cox-1 prevent luteolysis and therefore the fall in circulating progesterone, thereby explaining the delay in parturition^29^. However, recent work from our group has shown that the uterus contracts normally in Cox-1 null mice^30^, leaving the reason for parturition delay unanswered. Herein, we hypothesized that abnormal cervical maturation, rather than impaired uterine contractility, plays a key role in the parturition delay of Cox-1 knockout (KO) mice.

To investigate cervical remodeling in Cox-1 KO mice throughout parturition, we employed a non-invasive method capable of probing the cervical microenvironment longitudinally *in vivo*. In the past decade, numerous innovative cervical assessment tools that span optical, acoustic, electrical, and direct mechanical approaches have been developed^2, 31, 32^. These tools have already improved our understanding of cervical remodeling in term and preterm birth. A few of these techniques are directly sensitive to biochemical composition. Fluorescence spectroscopy has been used to measure collagen concentration and solubility in women and animal models^33, 34^, which has consistently shown decreasing fluorescence intensity throughout pregnancy. Diffuse reflectance spectroscopy has measured significant increases in water and decreases in hemoglobin after application of prostaglandins for cervical ripening^35^, as well as changes in optical properties based on hormonal status^36^ and stage of pregnancy^37^. Second harmonic generation, an optical imaging method that probes fibrillar collagen, has confirmed increasing collagen dispersion throughout pregnancy in *ex vivo* human^38^ and mouse studies^39, 40^. More recently, optical coherence tomography was used to measure the three dimensional structure of collagen fibrils in the human uterine cervix, and found higher dispersion in excised pregnant tissues than non-pregnant samples^41^. These studies have yet to be performed *in vivo*, but contribute to increasing our understanding of collagen’s role in cervical remodeling during pregnancy.

In this paper, we present the application of Raman spectroscopy, a real time light-based approach that probes biochemical information of tissues *in vivo*, to study the pregnant cervix. Raman spectroscopy measures inelastically scattered photons generated by light interaction with molecular bonds. Each molecule has specific vibrational frequencies, and Raman-scattered light can be analyzed to determine the type of molecules present in any target tissue. Raman spectroscopy is sensitive to proteins, water, nucleic acids, lipids, and carbohydrates, and measures these markers simultaneously with high specificity^42–45^. Raman spectroscopy was first applied to the *in vivo* cervix in the context of cervical dysplasia detection^46^. While conducting optimization of cervical dysplasia detection algorithms, it was discovered that Raman spectroscopy is sensitive to the effects of hormonal status on healthy cervical tissue^47–49^. These findings led to the investigation of longitudinal biochemical changes that can be measured by Raman spectroscopy in the cervix during pregnancy *in vivo*, both in mouse models and human subjects^31, 50^.

The goal of this study was to non-invasively test the hypothesis that cervical remodeling, rather than uterine contractility, is impaired in Cox-1 KO mice and contributes to their delayed parturition phenotype. *In vivo* Raman spectroscopy was used to determine whether biochemical differences exist in the cervix of Cox-1 KO mice during pregnancy compared to wild type (WT). Additionally, we examined whether Raman spectra could be correlated with changes in biomechanical properties of the cervix including stiffness and distensibility in WT and Cox-1 KO mice. Finally, *ex vivo* biochemical assays were performed to validate results obtained from *in vivo* Raman spectroscopy.

## Results

### The uterus contracts normally during delayed parturition in Cox-1 KO mice

**Figure 1:**
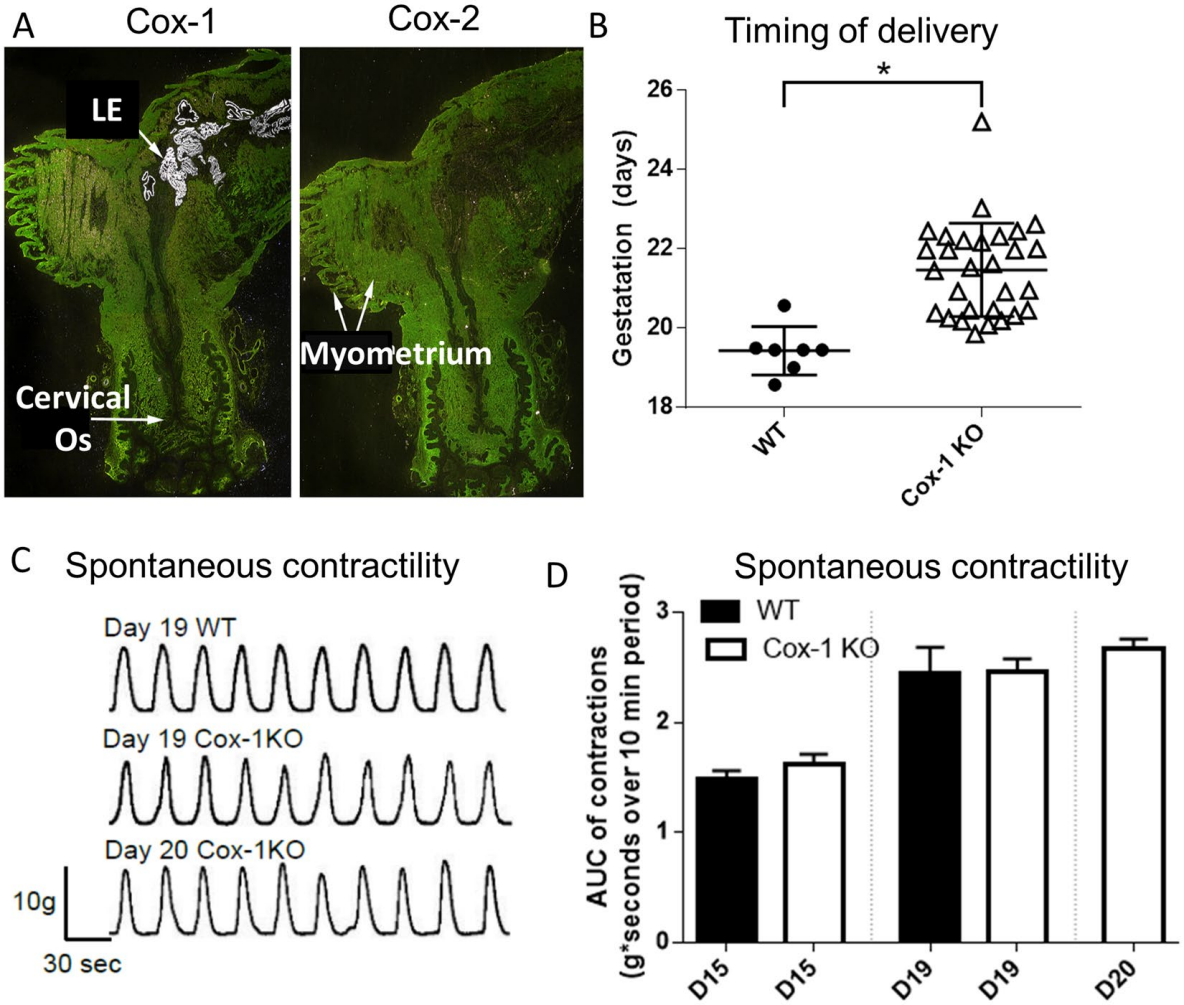
Cox-1 expression in the pregnant mouse uterus, and the effect of global Cox-1 deletion on parturition and uterine contractility. (**A**) *In situ* hybridization of 35S-labeled Cox-1 and Cox-2 in WT day 19 mouse uterus. (**B**) Timing of delivery was recorded from pregnant WT and Cox-1 KO mice (*p = 0.0001). (**C**) Representative *ex vivo* contractility tracings of myometrial strips from pregnant mice on the indicated days of pregnancy. (**D**) Recordings of myometrial contractility (n = 5–11 mice per group) were analyzed for area under the curve (AUC). Mean ± SEM.

Examination of Cox-1 (*Ptgs1*) and Cox-2 (*Ptgs2*) messenger ribonucleic acid (mRNA) expression on the day of expected delivery (day 19) in WT mice (Fig. 1A **)** revealed strong Cox-1 expression in the uterine luminal epithelium (LE) but low expression in the cervix; Cox-2 expression was low throughout the lower uterine segment. The timing of delivery was significantly delayed (p = 0.0001) in Cox-1 KO compared to WT mice (Fig. 1B). Despite strong Cox-1 expression in the uterus, representative tracings from *ex vivo* spontaneous uterine contractility experiments show that contraction patterns (Fig. 1C), and contractile activity (area under the curve of contractions per unit time) did not differ between WT and Cox-1 KO uteri on gestation day 15, 19, or comparison of WT day 19 versus Cox-1 KO day 20 (Fig. 1D). Thus, normal uterine function during delayed parturition suggests that the cervix may contribute to the delay, providing motivation for studying cervical remodeling in this model.

### Raman spectral signatures from wild type and Cox-1 KO cervices deviate at the time of parturition

**Figure 2:**
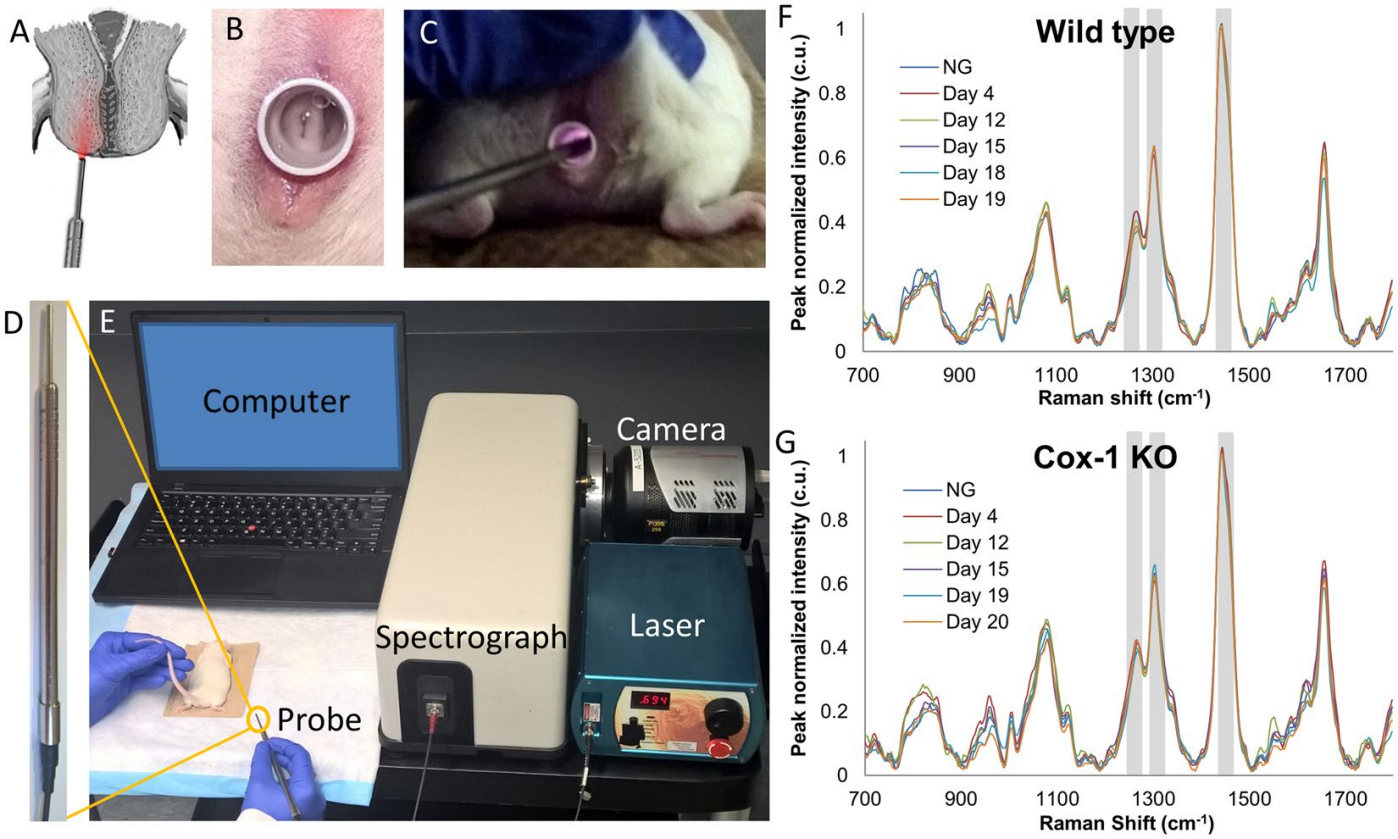
*In vivo* Raman spectroscopy system for cervical assessment during pregnancy. (**A**) Illustration showing the placement of the Raman spectroscopy fiber optic probe against the ectocervix of the mouse. (**B**) Mouse cervix visualized using a speculum. (**C**) *In vivo* Raman spectroscopy during measurement of mouse cervix. (**D**) Raman spectroscopy fiber optic probe. (**E**) Image of *in vivo* Raman spectroscopy system. Average Raman spectra from different time points during pregnancy in WT (**F**) and Cox-1 KO (**G**) mice. Gray boxes indicate regions that were highlighted in Figs 3–4.

**Figure 3:**
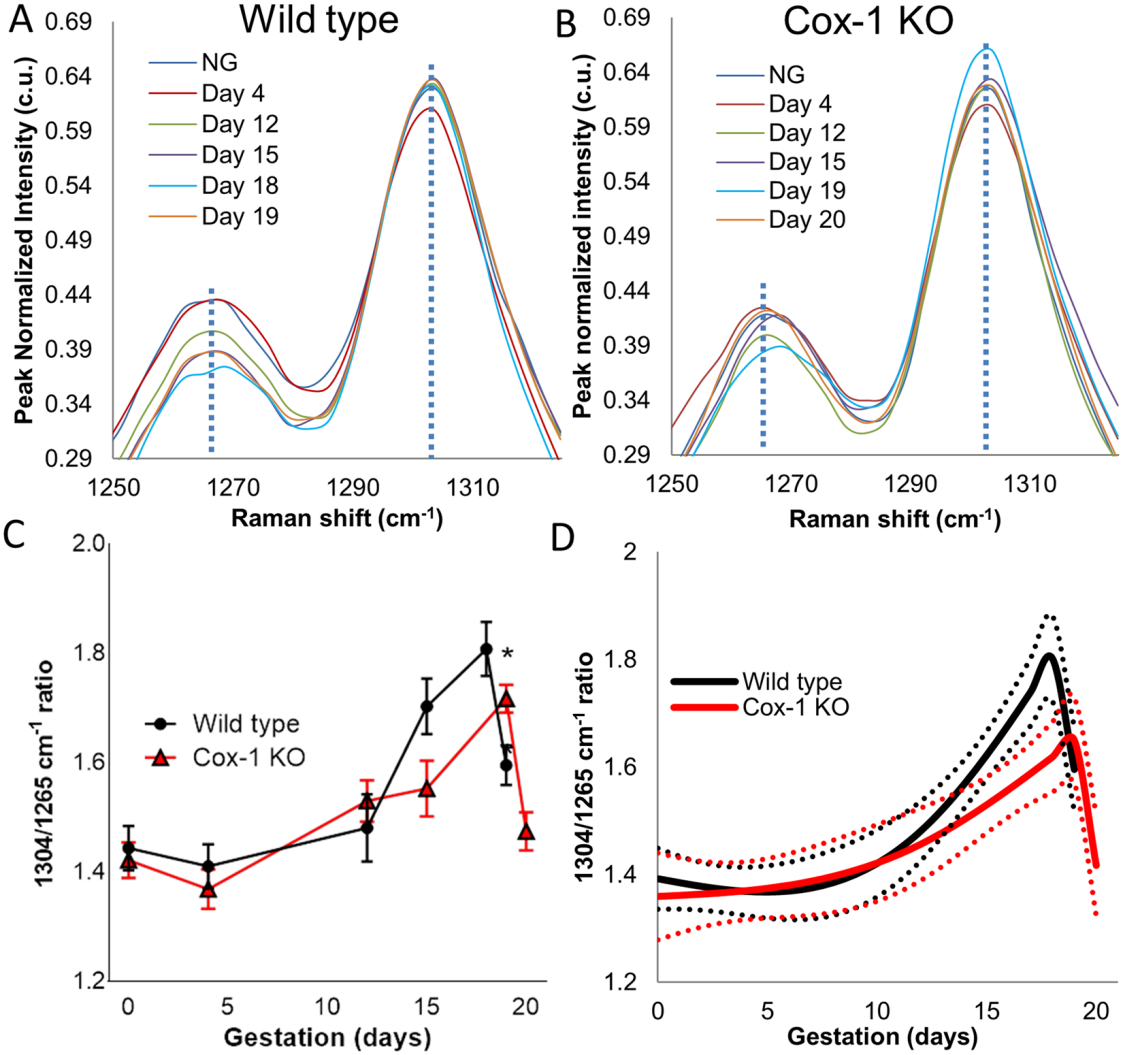
Raman spectral bands change with pregnancy and are delayed in the Cox-1 KO mouse compared to WT. Average Raman spectra from the 1265 cm^−1^ peak (blue dashed line) and 1304 cm^−1^ peak (blue dashed line) during different time points of pregnancy in WT (**A**) and Cox-1 KO (**B**) mice. (**C**) Mean ± SEM of the 1304 cm^−1^ to 1265 cm^−1^ peak ratio as a function of gestation in WT and Cox-1 KO mice (p < 0.05). (**D**) Modeled longitudinal trajectories of the 1304 cm^−1^ to 1265 cm^−1^ Raman peak ratio.

**Figure 4:**
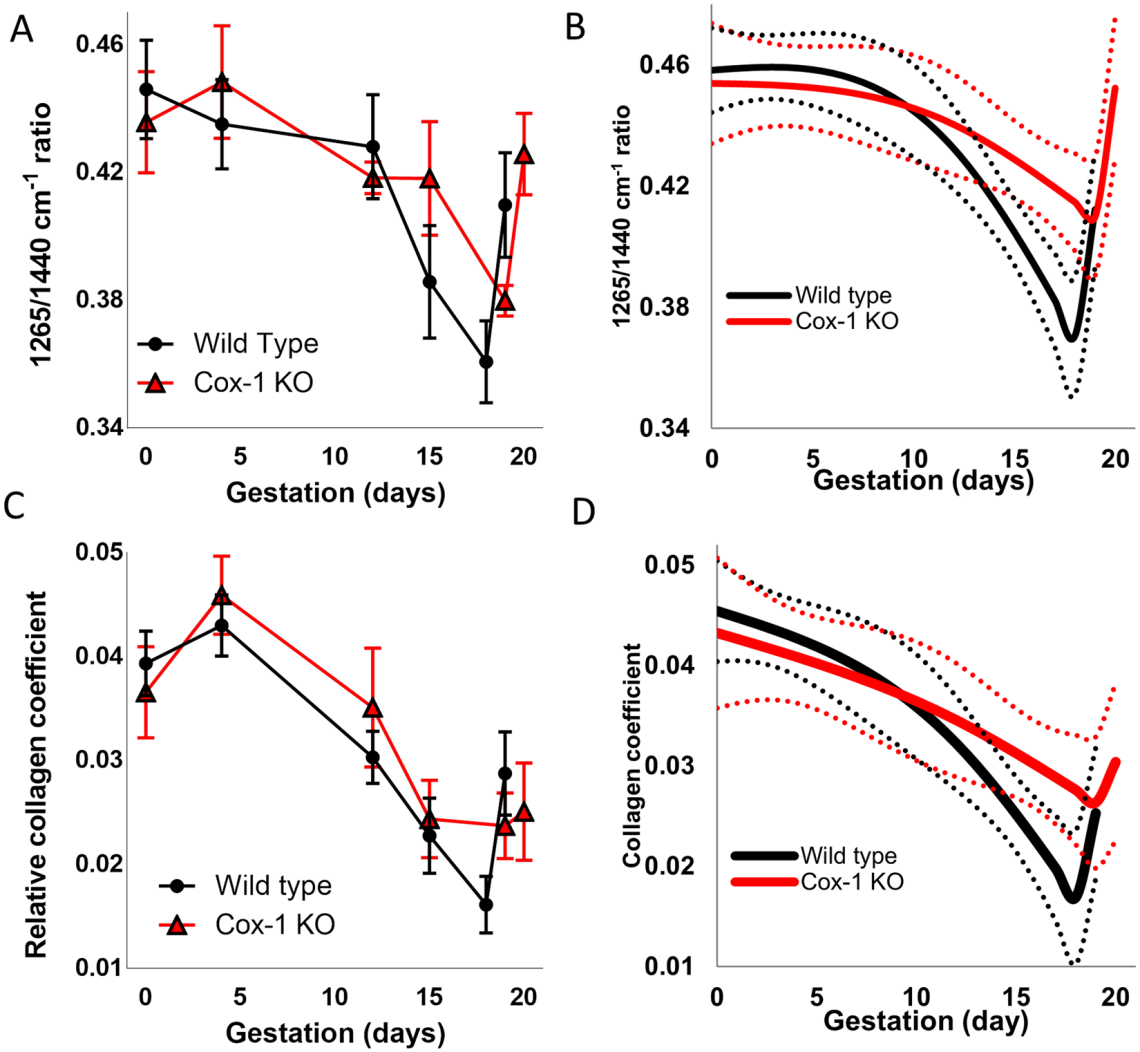
Raman spectra reveal delayed remodeling in the Cox-1 KO mouse at term. Mean ± SEM (**A**) and modeled longitudinal trajectories (**B**) of the 1657 cm^−1^ to 1440 cm^−1^ peak ratio. Mean ± SEM (**C**) and modeled longitudinal trajectory (**D**) of the non-negative least squares model collagen coefficient as a function of gestation in WT and Cox-1 KO mice.

An approach for using Raman spectroscopy to assess the biochemical status of the cervix over time in WT and Cox-1 KO mice was developed. Raman spectra were acquired *in vivo* from the cervix of non-gravid (NG) and pregnant WT and Cox-1 KO mice using a portable Raman spectroscopy system (Fig. 2A–E). Measurements were taken from multiple sites on the ectocervix with the aid of a small speculum (Fig. 2B,C) for visualizing the probe location. A custom-built fiber optic Raman probe was used for spectral acquisition and a 3 second integration time was used for each spectrum. Average peak-normalized spectra (normalized to 1440 cm^−1^ peak for spectral visualization purposes only, all subsequent statistical analysis was performed on non-normalized spectra) were plotted for each mouse model over the course of pregnancy (n = 8–10 mice per group, per time point) (Fig. 2F,G). Regions of interest (gray bands, Fig. 2F,G) that showed maximum differences were identified for further analysis (Figs 3–4).

Ratios of multiple Raman peaks were calculated and plotted as a function of gestation day and mouse model to quantitatively evaluate spectral changes over time. Of the various peaks analyzed, the 1304 cm^−1^ peak increased while the 1265 cm^−1^ peak decreased throughout gestation (dashed lines in Fig. 3A,B). The 1304 cm^−1^ to 1265 cm^−1^ Raman peak ratio tentatively assigned to lipid/protein change^42, 43^ increased starting at day 12 in both mouse models, however, the rate of change was higher and reached a larger ratio in the WT compared to the Cox-1 KO (Fig. 3C) (p < 0.05). Furthermore, a sharp decrease was observed in the WT on day 19 of pregnancy (p < 0.05), but this decrease did not occur in the Cox-1 KO until day 20 (Fig. 3C). The trends seen in other Raman peaks at 1003 cm^−1^ (phenylanaline^42^), 1440 cm^−1^ (lipids^43^), and 1657 cm^−1^ (proteins^42^) are displayed in Supplementary Fig. S1. To test the generalizability of the Raman spectral changes measured over pregnancy, as well as provide curve analysis and interpolation between measured points, a generalized linear model was developed (Fig. 3D). The 1304/1265 cm^−1^ peak ratio model showed similar features to the data presented in Fig. 3C, and revealed highly significant changes as a function of gestational age (p < 0.0005) and mouse model (p < 0.0005).

Besides 1304/1265 cm^−1^, multiple peak ratios displayed significantly different trajectories over the course of pregnancy between WT and Cox-1 KO mice. Figure 4A shows the trajectory of the 1265 cm^−1^/1440 cm^−1^ ratio which gradually decreased until day 12 in both mouse models. The WT showed a steeper decrease from day 12 to day 18, but then sharply increased on day 19. The Cox-1 KO did not decrease as steeply and not until day 15 before sharply increasing on day 20. The three phases of spectral change may be analogous to the softening, ripening and dilation phases^3^ in WT and Cox-1 mice but with a delay in the ripening to dilation transition in the Cox-1 KO mice. The 1265/1440 cm^−1^ ratio model showed significant changes over the course of pregnancy (p < 0.0005) and as a function of mouse model ((p < 0.0005), as well as in the interaction between gestation and mouse model (p < 0.05) (Fig. 4B).

A non-negative least squares (NNLS) analysis was performed to understand the biochemical basis of the *in vivo* Raman spectra. Pure spectra were collected from collagen I, water, and adipose tissue which revealed characteristic fingerprints, demonstrating molecular specificity (Supplementary Fig. S2). Although adipose tissue is not known to be present within the cervix, it is likely that surrounding fat pads such as the inguinal, perigonadal, and lower pelvic fat pads were detected in some of the Raman spectra^51^, and as such adipose was incorporated into the NNLS model. A representative cervical tissue spectrum (black) was fit using the NNLS model (red) developed using the pure component spectra, and then overlaid with the residual (blue) (Supplementary Fig. S2). The residual was small and fluctuated above and below zero. Furthermore, the least squares fit was decomposed such that the contribution from each component was easily seen (Supplementary Fig. S2). The contribution of collagen in Raman signals decreased over the course of pregnancy starting on day 12 in WT and Cox-1 KO mice. WT levels steadily declined until day 18 and then spiked on day 19, but Cox-1 KO levels plateaued at day 15 and increased on day 19 and 20 (Fig. 4C). The collagen coefficient was also analyzed using the developed generalized linear model (Fig. 4D), and revealed significant changes over gestation (p < 0.0001), but no significant differences were reported between mouse models. To verify this result without the influence of adipose tissue, the cervix was excised from non-gravid and day 19 WT mice and Raman spectra were obtained. The spectra were fit to the NNLS model which revealed that the collagen coefficient was lower on day 19 of pregnancy (Supplementary Fig. S3), similar to the *in vivo* results.

### Wild type and Cox-1 KO *ex vivo* cervical biomechanical properties deviate at the end of pregnancy

**Figure 5:**
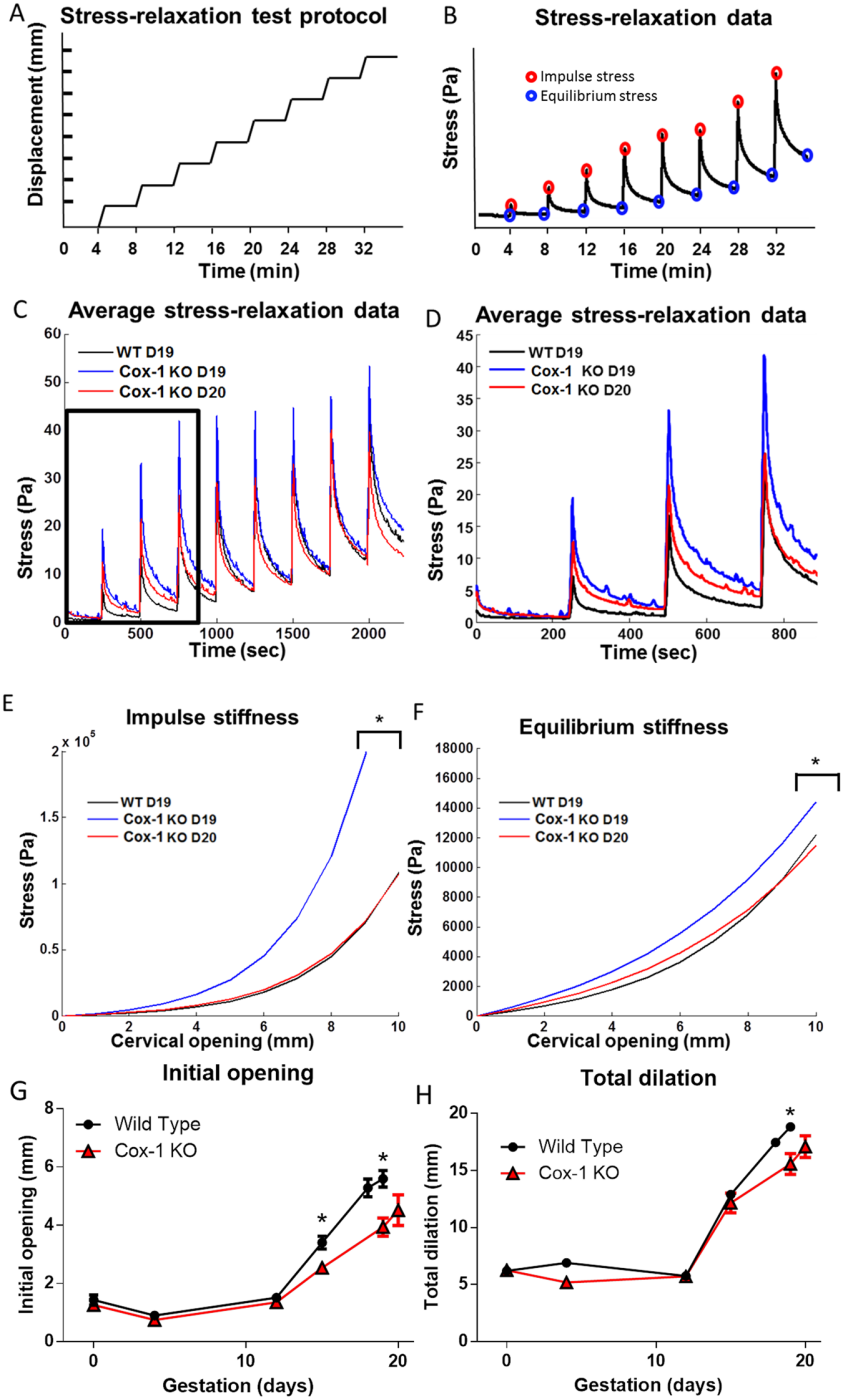
Cox-1 KO mice have less distensible cervices at term than WT. (**A**) Displacement protocol used for biomechanical stress relaxation tests. (**B**) Representative data from stress-relaxation biomechanical tests. Red circles highlight the maximum impulse stress observed for each displacement; blue circles highlight the equilibrium stress observed four minutes post displacement. (**C**) Average stress-relaxation recordings from WT day 19, Cox-1 KO day 19, and Cox-1 day 20 cervical tissues. (**D**) Inset of the average stress-relaxation data from the first three displacements. (**E**,**F**) Average exponential fits to the impulse stiffness and equilibrium stiffness results from WT day 19, Cox-1 KO day 19, and Cox-1 KO day 20 cervical tissues, *p < 0.05 compared to day 19 WT. (**G**) Unloaded dilation of *ex vivo* cervical tissues from WT and Cox-1 KO mice across gestation (n = 8–10 per group, per time point). (**H**) Total dilation of *ex vivo* cervical tissues prior to tissue failure.

After performing *in vivo* Raman spectroscopy, the cervices of select mice (n = 8–9 per group, per time point) were excised for paired *ex vivo* biomechanical testing to determine whether mechanical properties changed in response to altered tissue biochemistry with pregnancy. A defined tissue displacement protocol was followed for biomechanical testing (Fig. 5A). Representative data from a WT day 15 cervix that underwent stress-relaxation testing is shown in Fig. 5B; red circles indicate the impulse stress experienced by the tissue for each increase in displacement and provides information regarding the elastic nature of the tissue. Blue circles indicate the equilibrium stress that cervical tissues maintained after a strain, providing information on viscous properties.

Average raw data generated from WT day 19, Cox-1 KO day 19, and Cox-1 day 20 mice segregated into distinct groups (Fig. 5C,D). Over the first three displacements performed (Fig. 5D), Cox-1 day 19 tissues experienced the greatest amount of stress per millimeter of displacement, followed by the Cox-1 day 20 tissues, and then WT day 19 tissues, which exhibited a smaller degree of stress, particularly at the first two displacements.

The impulse and equilibrium stress were modeled using an exponential curve described by Timmons *et al*.^21^ to compare the stiffness of the cervical tissues in WT and Cox-1 KO mice. The stresses were significantly greater in the gestation day 19 Cox-1 KO tissues compared to day 19 WT and day 20 Cox-1 KO (Fig. 5E,F). The A and B coefficients were significantly higher in Cox-1 KO day 19 tissues compared to WT day 19 and Cox-1 KO day 20 (Table 1, p < 0.05), indicating that the Cox-1 KO tissues experienced greater stress at small and large displacements, respectively. Taken together, these data (Fig. 5A–F) indicate that the Cox-1 KO cervix is more rigid at term, but becomes more like the compliant WT cervix after one additional day of gestation.

Initial dilation of the cervical os measured in excised, unloaded tissues was significantly different (p < 0.05) between time-matched WT and Cox-1 KO mice on gestation day 15 and 19, as well as between day 18 WT and day 19 Cox-1 KO (Fig. 5G). However, day 19 WT compared to day 20 Cox-1 KO was not significantly different, indicating catch-up dilatability of the post-mature Cox-1 KO cervix under baseline conditions. Total cervical dilation (initial dilation + displacement at failure during stress-relaxation testing) was measured across gestation (Fig. 5H). On day 19 of pregnancy, the WT cervix was able to dilate to a greater extent than Cox-1 KO before tissue failure occurred (p < 0.05). No difference was observed between the day 19 WT and day 20 Cox-1 KO cervices, suggesting eventual catch-up of KO tissues as the parturition delay continued.

### *In vivo* Raman spectra correlate with *ex vivo* biomechanical measures

**Figure 6:**
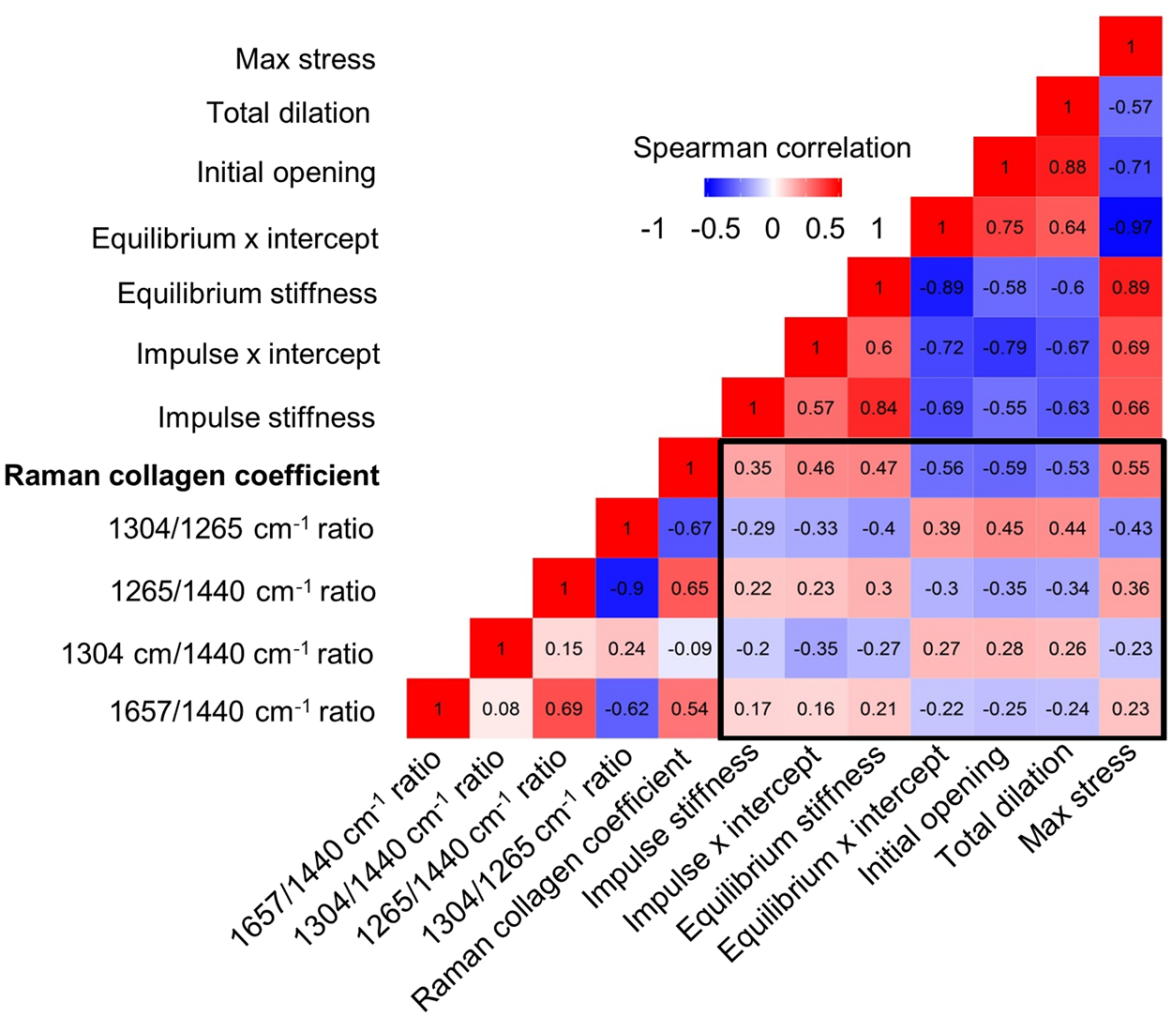
*In vivo* Raman data correlate with *ex vivo* biomechanical properties. Spearman correlation matrix containing four Raman peak ratios and a non-negative least squares component representative of collagen signatures for comparison to the impulse stiffness, impulse x intercept, equilibrium stiffness, equilibrium x intercept, initial opening, total dilation, and maximum stress measures obtained during biomechanical testing.

Multiple Spearman correlations were computed between Raman spectral features and mechanical measures (Fig. 6). The black box highlights correlations between Raman spectra and biomechanical properties of the cervix where strong positive correlations (bright red) and strong negative correlations (bright blue) were observed. Many of these comparisons were statistically significant, with the highest correlations coming from the Raman spectral collagen coefficient (p < 0.005 for all biomechanical comparisons), followed by the 1304/1265 cm^−1^ lipid/protein ratio (p < 0.05 for all biomechanical comparisons).

### Cervix progesterone levels remain elevated in Cox-1 KO mice at the end of pregnancy

**Figure 7:**
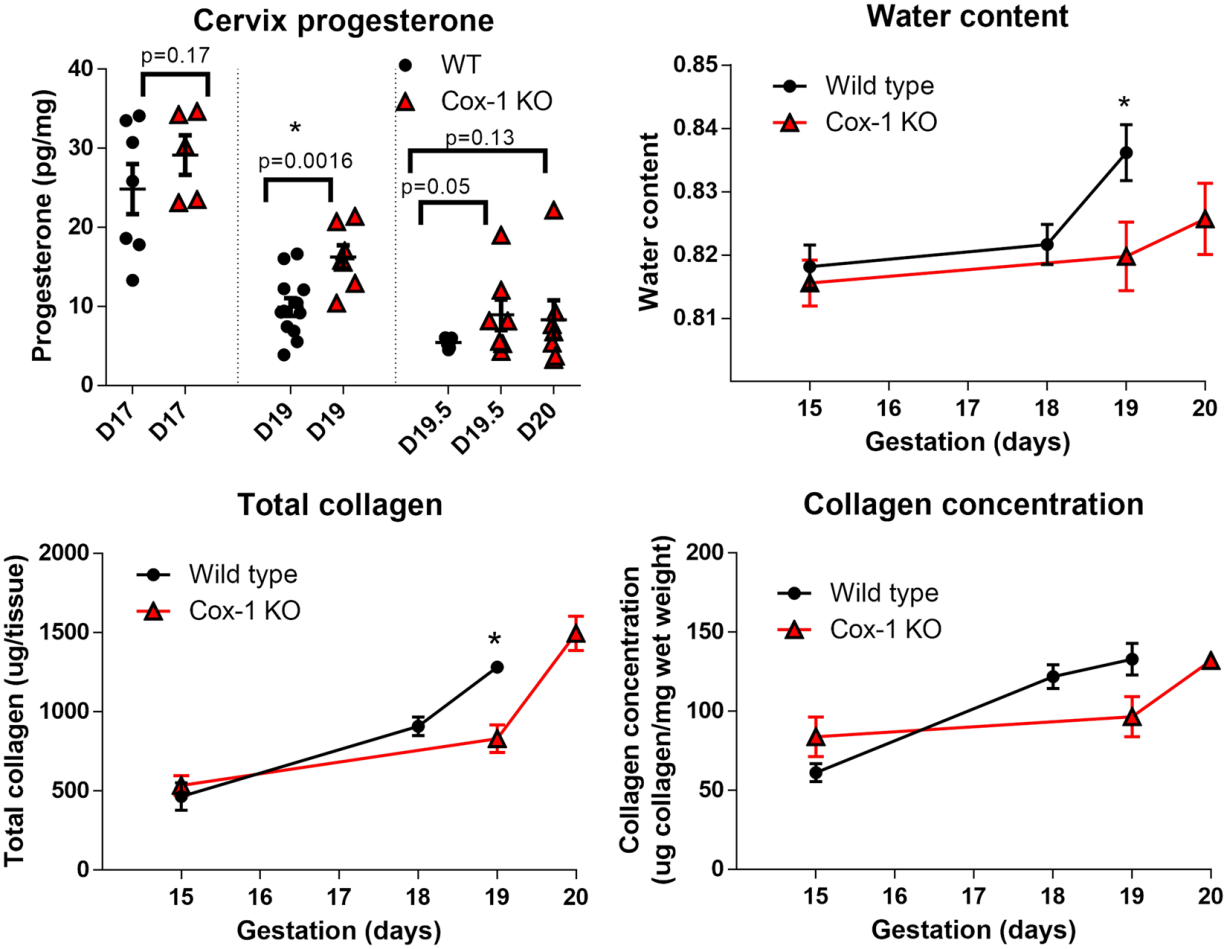
*Ex vivo* biochemical assays of cervix composition show delayed remodeling and Cox-1 KO mice. (**A**) Cervix progesterone levels. (**B**) Water content. (**C**) Total collagen. (**D**) Collagen concentration (μg/mg wet weight). Plotted as mean ± SEM, *p < 0.05 compared to day 19 WT.

Progesterone levels are elevated during pregnancy to maintain a quiescent state in mice. Cervical tissue progesterone levels were measured using radioimmunoassay detection in WT mice on gestation day 17 to determine progesterone levels prior to the onset of labor, on gestation day 19 to measure pre-parturition levels, and on day 19.5 to measure levels at the time of active labor. Similarly, Cox-1 KO cervical tissues were measured on gestation days 17, 19, and 19.5, as well as gestation day 20 due to delayed parturition (Fig. 7A). As expected, gestation day 17 progesterone values were the highest in both WT and Cox-1 KO pregnant mice since luteolysis had not yet occurred. On gestation day 19, progesterone levels declined in both WT and Cox-1 KO mice compared to day 17 (p < 0.001) although Cox-1 KO levels remained significantly elevated compared to WT (p < 0.005). On gestation day 19.5, progesterone levels were significantly lower (p < 0.05) in the WT group compared to time-matched Cox-1 KO mice. While Cox-1 KO mice still had higher mean progesterone levels on gestation day 20 compared to WT mice on gestation day 19.5, the difference was not statistically significant (p = 0.13).

### Tissue hydration and collagen content significantly differ at term gestation

In order to validate the results obtained from *in vivo* Raman spectroscopy, we performed *ex vivo* biochemical assays to measure tissue hydration and collagen content in cervix samples from WT and Cox-1 KO mice. Gestation day 19 WT mice had significantly greater water content than Cox-1 KO mice on day 19 (p < 0.05) but not day 20 as indicated by these assays (Fig. 7B). An established collagen assay based on hydroxyproline content was used to determine the total collagen content (micrograms of collagen per tissue) (Fig. 7C). Total collagen slowly increased from day 15 within both WT and Cox-1 KO mice. However, significantly higher (p < 0.01) levels of collagen were detected in WT mice on day 19, which was eventually achieved in the Cox-1 KO mice on day 20 of gestation. No significant differences were observed between WT and Cox-1 KO cervical tissues in collagen concentration (Fig. 7D) for any time points examined.

## Discussion

Prostaglandins are key regulators of multiple aspects of female reproduction, including uterine contractility, and cervical ripening in inflammation-induced preterm birth^21, 52, 53^. Impaired luteolysis and prevention of the expected progesterone decline in Cox-1 KO mice is currently considered the mechanism for delayed parturition in these mice^29^ and similar models of prolonged gestation^15, 29, 54–58^. Despite speculation of suppressed uterine contractility of Cox-1 KO mice, we observed unhindered uterine contractility despite a 2–3 day delay in delivery of offspring, indicating that uterine contractility is not the basis for delayed parturition and suggests that impaired cervical remodeling may be responsible for preventing normal birth timing^30^. We used Raman spectroscopy to show for the first time that parturition-related changes in biochemical composition of the cervix are delayed in Cox-1 mice. This optical technique correlates to biomechanical measures of cervical rigidity and can be used to non-invasively stage maturation of the cervix over the course of pregnancy and in the pre-parturition period leading up to birth.

Although prostaglandins are widely regarded as essential molecules for cervical ripening, our data showing restricted Cox expression in the pregnant mouse cervix corroborate recent unexpected findings by Timmons *et al*.^21^ where low levels of Cox-1 and Cox-2 expression and low cervical prostaglandin levels were noted during normal pregnancy. Cervix prostaglandin levels were increased in the immediate post-partum period, likely related to heightened Cox expression or via prostaglandin synthesis in nearby uterine tissues (Fig. 1), which then bind and activate cervix prostaglandin receptors^59^. While upregulation of Cox-2 can partially compensate for the absence of Cox-1 in the uterus^60^, we previously showed that prostaglandin levels were at the lower limits of detection in Cox-1 KO uteri^12^. Interestingly, our present study showed that deletion of Cox-1 did not impair uterine contractions (Fig. 1C,D). It was previously thought that Cox-1-derived prostaglandins directly stimulate myometrial contractility or that PGF2α acts on the corpus luteum, leading to subsequent luteolysis, progesterone fall, and uterine activation for labor^61^. The compensatory mechanisms enabling normal uterine contractions in Cox-1 KO mice are not yet fully understood, but support an impaired cervical remodeling hypothesis that was tested in the current study.

*In vivo* Raman spectroscopy revealed significant changes in the biochemical makeup of the cervix over the course of pregnancy. Highly significant differences observed in Cox-1 KO compared to WT cervices near term provide the first evidence supporting impaired cervical remodeling as a novel factor responsible for the parturition delay in Cox-1 KO mice. In particular, Cox-1 KO mice show a gradual increase in the 1304 cm^−1^/1265 cm^−1^ peak ratio compared to a non-linear increase in the WT peak ratio over pregnancy, with a sharp decrease at term in WT and one day after term in Cox-1 KO (Fig. 3). The peak at 1265 cm^−1^ decreased linearly over pregnancy, which is assigned to the Amide III vibration (C-N stretching and N-H in-plane bending from peptide backbone) present in various structural proteins, as well as in-plane cis double bond =C-H bending from lipid molecules such as adipose tissue and phospholipids^43, 62^. The 1304 cm^−1^ peak is assigned to scissoring and twisting vibrations of CH_3_ groups in lipids^43^, but also has overlap with spectral bands in actin, elastin, and nucleic acids^42, 45, 63, 64^. Thus, this ratio is tentatively assigned as a lipid-to-protein ratio and may be indicative of an increase in collagen disorganization relative to cellular content in the softening and ripening stages of pregnancy.

A reduced rate of change in the 1265 cm^−1^/1440 cm^−1^ ratio was observed in the ripening phase in Cox-1 KO mice compared to WT (Fig. 4A,B). The 1440 cm^−1^ peak is characteristic of CH_2_/CH_3_ scissoring in lipids^43^. Collagen and other extracellular matrix proteins break down over the course of pregnancy^4^ and are likely responsible for the observed decrease in this ratio as pregnancy advanced. Significant changes over the course of pregnancy were also noted in the NNLS collagen coefficient (Fig. 4C,D), where both WT and Cox-1 KO levels significantly decreased starting on day 12 of pregnancy. The collagen concentration is known to decrease during pregnancy^8, 65^, which aligns with results from both the *in vivo* and *ex vivo* Raman spectra. In contrast to the peak ratios, the Cox-1 KO collagen coefficient did not sharply rise at term, nor were significant differences found between WT and Cox-1 KO mice at term, indicating that sharp changes observed prior to parturition in Figs 3 and 4A,B may not be centered on collagen. It should be noted that although the pure components measured for the developed NNLS model account for a large amount of the variability observed in the collected data set, not all of the potential biochemical contributors to the measured Raman signal are incorporated. Additional cervical components include but are not limited to actin, elastin, glycosaminglycans, and nucleic acids^3, 66, 67^, and likely contribute to the sharp changes observed prior to delivery in both WT and Cox-1 KO mice in the Raman peak ratios (Figs 3C and 4A), and WT mice in the NNLS analysis (Fig. 4C).

Differences observed in Raman spectra were supported by *ex vivo* biomechanical tests (Fig. 5), which showed that the cervix of day 19 Cox-1 KO mice had significantly higher impulse stiffness and equilibrium stiffness compared to day 19 WT. Possible explanations for the impulse stiffness include higher levels of collagen crosslinks in the Cox-1 KO tissue at term^4^, whereas less dispersion of collagen fibers and less hydrated cervical stroma could prevent viscous relaxation resulting in higher equilibrium stiffness^68, 69^. Furthermore, Cox-1 KO day 19 tissues were less compliant in both unloaded states (initial dilation) as well as the maximum stretch-induced dilation state (total dilation or ultimate cervical compliance) compared to day 19 WT cervices. The latter two measures offer complementary information. Initial dilation reflects tissue compliance without application of any external force (other than gravity) similar to cervical dilation assessment in non-laboring women. For example, pregnant women with cervical dilation <3 cm require additional force to produce dilation sufficient for parturition compared to women dilated to 8 cm (maximum of 10 cm for complete dilation), even if both patients have an equally softened cervix. Although the *in vivo* cervix is always under load^70^, our experiments isolated the cervix from surrounding tissue forces and pressure from the fetus to provide information on inherent cervical distensibility. Total dilation, which yields the maximum stretch cervical tissue can withstand prior to tissue failure, is similar to the requirement for human cervical dilation to 10 cm. Regardless of cervical diameter at the onset of labor, delivery will not be successful until complete dilation is achieved, and is therefore an absolute, not relative requirement for parturition. Together, this information confirms that normal transition to the cervical dilation phase was delayed in Cox-1 KO mice, resulting in reduced capacity to reach the final dilation needed to successfully deliver at term.

Successful passage of a fetus depends on achievement of cervical remodeling and complete cervical dilation. Currently, physical and biomechanical assessments are the most commonly used measures of cervical remodeling. Here, we showed that Raman peak ratios and changes in biochemical composition of the cervix correlate with biomechanical properties and physical changes in cervical stiffness and compliance (Fig. 6). The highest correlation observed was between the Raman collagen coefficient and the initial dilation. Fibrillar collagens largely govern the mechanical properties of cervical tissue, supporting the feasibility of using Raman measurements to interrogate cervical maturation during the quiescent phase of pregnancy and with impending parturition. Biochemistry is known to play a key role in mechanical properties^68, 69, 71^; however, biochemistry alone cannot fully capture the structure and packing of all molecules that contribute to biomechanical properties. As such, pairing Raman spectroscopy with another structurally-focused modality could greatly improve the determination of mechanical properties *in vivo*. Engineering models of cervical biomechanics^72^ and biochemical inputs determined via Raman spectroscopy could be valuable additions when applying such models towards non-invasive assessment of cervical tissue.

*Ex vivo* biochemical assays similarly revealed differences between WT and Cox-1 KO cervical tissues at the end of pregnancy. Significantly higher progesterone levels were noted in Cox-1 KO mice on day 19 compared to WT, however unlike previous reports^29^, progesterone levels clearly fell in both groups, albeit not to the same extent. This suggests the possibility of a progesterone-mediated delay in cervical dilation, similar to two other mouse models of delayed parturition in which systemic progesterone levels remain elevated: Steroid 5a-reductase type-1 knockout mice and Tg/Tg mice with an insertion in chromosome 6^54, 57^. Water content rose significantly on the final day of pregnancy in WT mice, which was not observed in the Cox-1 KO day 19 tissues. Cervical hydration typically increases over the course of pregnancy, particularly in the ripening/dilation phase^6–8^. Similarly, total collagen per cervical tissue rose significantly on the final day of gestation in WT mice compared to Cox-1 KO, however collagen levels in the day 20 Cox-1 KO cervix eventually caught up to day 19 WT levels (Fig. 7C).

One limitation of this study is the contribution of adipose tissue to the *in vivo* Raman spectra collected from the cervix. Multiple steps were taken to minimize the influence of adipose tissue whilst maintaining information from the cervix, including an automated system for removing spectra that had high adipose content (spectra with the 1440 cm^−1^ peak >1800 counts). In addition, the generalized linear model incorporates the 1440 cm^−1^ signal intensity as a model variable so that the non-adipose content could be independently analyzed. This allowed for prediction of how the spectra would change over gestation given a constant adipose contribution. This method can be used for other applications where an additional signal is interfering with spectra from a desired location, including interference from adipose tissue and bone. Beyond quantitative data exclusion (by pre-determined criteria) and post-processing analysis, a variety of hardware solutions could be used to minimize the variability introduced by adipose. Use of a custom Raman fiber optic probe that is focused to superficial tissue depths could reduce contribution from deep adipose layers^73^. Furthermore, a spatially offset probe design could provide depth-resolved information with minimal signal from adipose tissue reaching the low source-detector distance fibers^74^. Finally, use of a lower wavelength such as 680 nm or 532 nm would inherently limit the penetration depth of the laser light, and therefore reduce contribution from adipose tissue located behind the cervix, however visible excitation sources are likely to lead to greater tissue heating and autofluorescence signal generation^75^.

In summary, we identified delayed cervical remodeling in pregnant Cox-1 KO mice as an underlying mechanism for prolonged gestation. Specifically, *in vivo* Raman spectroscopy detected significant differences between WT and Cox-1 KO cervical tissues during pregnancy, particularly in peaks representative of proteins and lipids. Spectral analysis correlated with biochemical changes, biomechanical testing, and reduced cervical dilation in Cox-1 KO mice at term. This study underscores the importance of studying the cervix in the context of abnormal parturition, which remains a relatively under-investigated organ. Difficulty obtaining molecular information from cervical tissue without compromising pregnancy has driven new innovation, including *in vivo* Raman spectroscopy. As a non-invasive, real-time technique that can be performed longitudinally, biochemical differences that occur throughout pregnancy can be detected without tissue injury or potential harm. Our study demonstrates that this technique, which has been used in a variety of human studies^76^, has clear potential for direct clinical translation.

## Methods

### Animals

All experiments involving animals were conducted in accordance with the regulations described in the NIH Guide for the Care and Use of Laboratory Animals, using protocols approved by Vanderbilt University Medical Center’s Institutional Animal Care and Use Committee (IACUC). Two groups of mice were investigated, CD-1 wild type (Jackson Laboratory) and Cox-1 null^28^. The Cox-1 null mouse was outbred to the CD-1 strain for 10 generations^12^. Animals were housed under a 12 hour dark-light cycle. Timed matings were conducted in the evenings and the presence of a post-copulatory plug the following morning defined gestation day 1. Wild type mice in this colony typically deliver on the evening of day 19 (designated d19.5). Six time points during pregnancy were investigated for each mouse genotype; WT mice were measured on days 4, 12, 15, 18, 19, and non-gravid, whereas Cox-1 KO mice were measured on days 4, 12, 15, 19, 20, and non-gravid. Non-gravid mice were measured in the diestrus phase of the estrous cycle as determined by vaginal lavage.

### *In situ* hybridization

Eleven micron thin frozen uterine sections were obtained from WT and Cox-1 KO mice and mounted on the same glass slides and *in situ* hybridization was performed as previously described^77^.

### *Ex vivo* myometrial contractility

Uterine myometrial strips were collected post-mortem, and used in an *ex vivo* isometric contractility assay as previously described^78^.

### Raman spectroscopy

A portable, fiber optic probe-based Raman spectroscopy system was used (Fig. 2E) for all Raman measurements acquired. The system consisted of an imaging spectrograph (Holospec f/1.8i, Kaiser Optical Systems, Ann Arbor, MI) coupled to a thermoelectrically cooled CCD camera (PIXIS: 256BR, Princeton Instruments, Princeton, NJ). A 785 nm diode laser (Innovative Photonic Solutions, Monmouth Junction, NJ) delivered 80 mW of power through a custom made fiber optic probe (Fig. 2D) (EmVision, Loxahatchee, FL) to the cervix.

The system was wavelength calibrated using a neon-argon lamp, and naphthalene and acetaminophen standards were used to determine the exact excitation wavelength for subsequent Raman shift calculations. The system was corrected for spectral response using a tungsten lamp that was calibrated by the National Institute of Standards and Technology. Spectra were smoothed using a Savitzky-Golay filter, background subtracted, and fluorescence subtracted using a modified polynomial fit method previously described^79^.

During measurements, all room lights were turned off. For *in vivo* experiments, animals were anesthetized using isoflurane, and the vaginal cavity and cervix were rinsed gently using saline. A small speculum tube was inserted to improve visualization of the mouse cervix and ensure contact of the fiber probe with the proper tissues during measurements (Fig. 2A–C). A range of 5–10 spectra were taken at multiple locations around the ectocervix of each mouse with an integration time of 3 seconds per measurement. For *ex vivo* experiments, the cervix was excised and immediately measured using an integration time of 10 seconds.

### Raman data analysis

Processed, non-normalized Raman spectra were used for data analysis. The signal to noise ratio (SNR) and adipose tissue signal from each Raman spectrum was calculated, and spectra with low signal (less than 100 counts at the 1440 cm^−1^ peak) as well as spectra with high adipose content (greater than 1800 counts at the 1440 cm^−1^ peak) were removed from analysis. Raman spectra were averaged by each mouse for display in Figs 2 and 3. Ratios from Raman peaks were calculated prior to averaging, and the results were subsequently averaged for each mouse. Peak ratios sensitive to ECM, lipids, and nucleic acids were plotted using the mean and standard error for each mouse model and gestation. Ratios were used as a way to normalize and make comparisons across many data sets that have varying levels of signal intensity and SNR. Student t-tests were performed to determine statistical significance (α = 0.05) between WT and Cox-1 KO mice at each gestation measured.

Raman spectra were modeled using generalized linear models in the R software, using the RMS package. The rate of change of various Raman peak ratios and NNLS coefficients over gestation were modeled and the WT and Cox-1 KO curves were compared. In this analysis, the Raman peak ratio or NNLS coefficient of interest was the dependent variable, whereas the gestation time and mouse model were independent variables. To account for signal intensity fluctuations, the spectral intensity (measured as the intensity of the 1440 cm^−1^ peak) was added as another independent variable which acts as a baseline offset. The time variable was modeled as a restricted cubic spline with three nodes to allow for non-linear behavior with respect to time. The last time point for each model (WT = 19, Cox-1 KO = 20) was assigned an indicator variable that allowed for the capture of significant changes at the end of pregnancy. The mouse model was set as a factor variable. To account for heteroscedasticity, the robust covariance function created in the rms package (‘robcov’) was used to adjust the standard errors. Finally, to evaluate interactions between the gestation time and mouse model, an ANOVA was performed on the generated curves from the developed regression model.

Non-negative least squares analysis was performed to determine the contributions of 3 different biochemical components as a function of gestation and mouse model^80–82^. This analysis follows the equation Y = β*X + ε, where the design matrix X was filled with pure biochemical spectra (collagen type I (Sigma), adipose tissue (collected from mouse abdomen post-mortem), and water), and the tissue spectrum being analyzed in Y. The optimum coefficients β that minimize the error ε were determined for each spectrum. For this analysis, NNLS regression was performed (MATLAB) such that components could not have a negative contribution on a spectral fit. For this study, the term “residual’ was defined as the difference between the “observed spectrum” and the “fitted spectrum”. A negative residual meant that in certain regions of the spectrum, the “fitted spectrum” was of greater intensity than the “observed spectrum.” None of the NNLS coefficients were calculated to be negative. The residuals per spectrum were summed and all spectra with a summed residual higher than 0.3 were removed from further analysis. The average coefficients for each biochemical component were calculated as a function of mouse model and gestation. Spearman correlations were performed on four Raman peak ratios and the collagen NNLS coefficient which were compared with biomechanical measures.

### *Ex vivo* biomechanical testing

Cervical tissue was excised post-mortem by cutting perpendicularly across the uterocervical junction where the uterine horns converge into the cervical canal. All vaginal tissue was removed and the dimensions of the cervical tissue were measured. The freshly excised cervical tissue was immediately placed in oxygenated Krebs Bicarbonate buffer at 37 °C for 10 minutes to equilibrate. Next, the tissue underwent stress-relaxation testing using a Radnoti Organ Bath System. This method allowed assessment of the viscoelastic behavior of each cervical tissue. The system was calibrated by recording force (measured in volts) with no added weight and with a 100 gram test weight, and the measured voltage was converted to force (mass x gravity). The tissue was then mounted on two stainless steel triangular hooks and attached to a force transducer with a mechanical drive. The hooks were slowly pulled apart until tension in the tissue was measured, after which the separation between the hooks was reduced to determine the “initial dilation” of the cervical tissue without strain-induced stress. This baseline distance (initial dilation) was recorded for each specimen, and then the stress-relaxation testing protocol was initiated as follows. The cervical tissue remained in an unloaded state for four minutes, and then experienced a strain of 0.1 mm/s for a total of ten seconds (one mm displacement), after which the strain was held constant for four minutes. This protocol was repeated until tissue failure, defined as the displacement at which sudden loss of stress is experienced by the tissue under tensile loading^83^, as shown in Fig. 5A. This process was controlled using stepper motors (Applied Motion Products) that were programmed with an Arduino Uno board. Recorded data was converted into stress based on the cross-sectional area of the tissues. Total dilation was calculated as the initial dilation plus displacement during biomechanical testing prior to tissue failure. Equilibrium stiffness was calculated for each tissue by plotting the average stress from the last 30 seconds of each stress cycle and generating a curve. The slope of the linear region of each curve was calculated as a simple measure of stiffness, as well as a model fit to the following exponential model described by Timmons *et al*.^21^:

$${\rm{Stress}}=({\rm{A}}/{\rm{B}})\ast [\exp ({\rm{cervical}}\,{\rm{opening}}\ast {\rm{B}})-1],$$

where the parameters A and B measure the stiffness at small and large displacements, respectively. The impulse stiffness was calculated for each tissue by plotting the maximum stress achieved from each stress cycle and generating a curve, which was similarly evaluated by taking the slope in the linear region, and a fit to the exponential model. Statistical analysis was performed on initial dilation, total dilation, equilibrium stiffness A and B coefficients, and impulse stiffness A and B coefficients using t-tests (*indicates p < 0.05).

### *Ex vivo* biochemical assays

For all *ex vivo* biochemical assays, cervical tissues were excised post-mortem, immediately flash frozen with Super Friendly FREEZE’IT (Fisher Scientific) and then placed in −80 C.

Progesterone concentrations were measured by the Endocrine Technologies Support Core (ETSC) at the Oregon National Primate Research Center (ONPRC, Beaverton, OR) using extraction-radioimmunoassay (RIA)^84^. Tissue samples were weighed and homogenized in 1 ml PBS, then extracted with 6 ml diethyl ether, dried under forced air stream and re-dissolved in assay buffer (0.1% gel-PBS). Progesterone values were corrected for extraction losses determined by radioactive trace recovery performed simultaneously with sample extraction, which ranged between 95.3–96.8% (n = 3). The assay range for the P4-extraction RIA was 5–750 pg/tube. The intra-assay variation for the assays ranged from 5.8–8.9% and inter-assay variation was 7.3%. Overall inter-assay variation for extraction-RIAs in the ETSC is less than 15%.

For cervical hydration and collagen content assays, tissues were weighed, and then lyophilized overnight. After freeze-drying, the tissues were weighed again. Based on these measurements, water content was determined as (wet weight/(wet weight + dry weight)). Next, a standard collagen extraction procedure was performed as previously described^8^. To quantify collagen content, a hydroxyproline assay was performed per manufacturer’s instructions (BioVision).

### Data Availability

The datasets generated and analyzed during the current study are available from the corresponding author on reasonable request.

## Electronic supplementary material

Supplementary Figures
