# Supplementary material for: In vivo Raman spectral analysis of impaired cervical remodeling in a mouse model of delayed parturition
Source: Sci Rep. 2017 Jul 28;7:6835. doi: 10.1038/s41598-017-07047-5 (PMC5533720; doi:10.1038/s41598-017-07047-5)
Supplement: Supplementary file 1 — Supplementary Figures [file 41598_2017_7047_MOESM1_ESM.pdf]

Title: *In vivo* Raman spectral analysis of impaired cervical remodeling in a mouse model of delayed parturition

Authors: Christine M. O'Brien[a], Jennifer L. Herington[b], Naoko Brown[b], Isaac J. Pence[a], Bibhash C. Paria[b], James C. Slaughter[c], Jeff Reese[a,b,d,1] Anita Mahadevan-Jansen[a,1*]

Author Affiliations:

a) Department of Biomedical Engineering, Vanderbilt University, Nashville, TN 37232

b) Department of Pediatrics, Vanderbilt University Medical Center, Nashville, TN 37232

c) Department of Biostatistics, Vanderbilt University Medical Center, Nashville, TN 37232

d) Department of Cell and Developmental Biology, Vanderbilt University Medical Center, Nashville, TN 37232

[1]J.R. and A.M.J are co-PIs of this work

*To whom correspondence should be addressed: Anita Mahadevan-Jansen, PO 351631 Station B, Nashville, TN 37235-1631 USA, 615-343-4787, anita.mahadevan-jansen@vanderbilt.edu

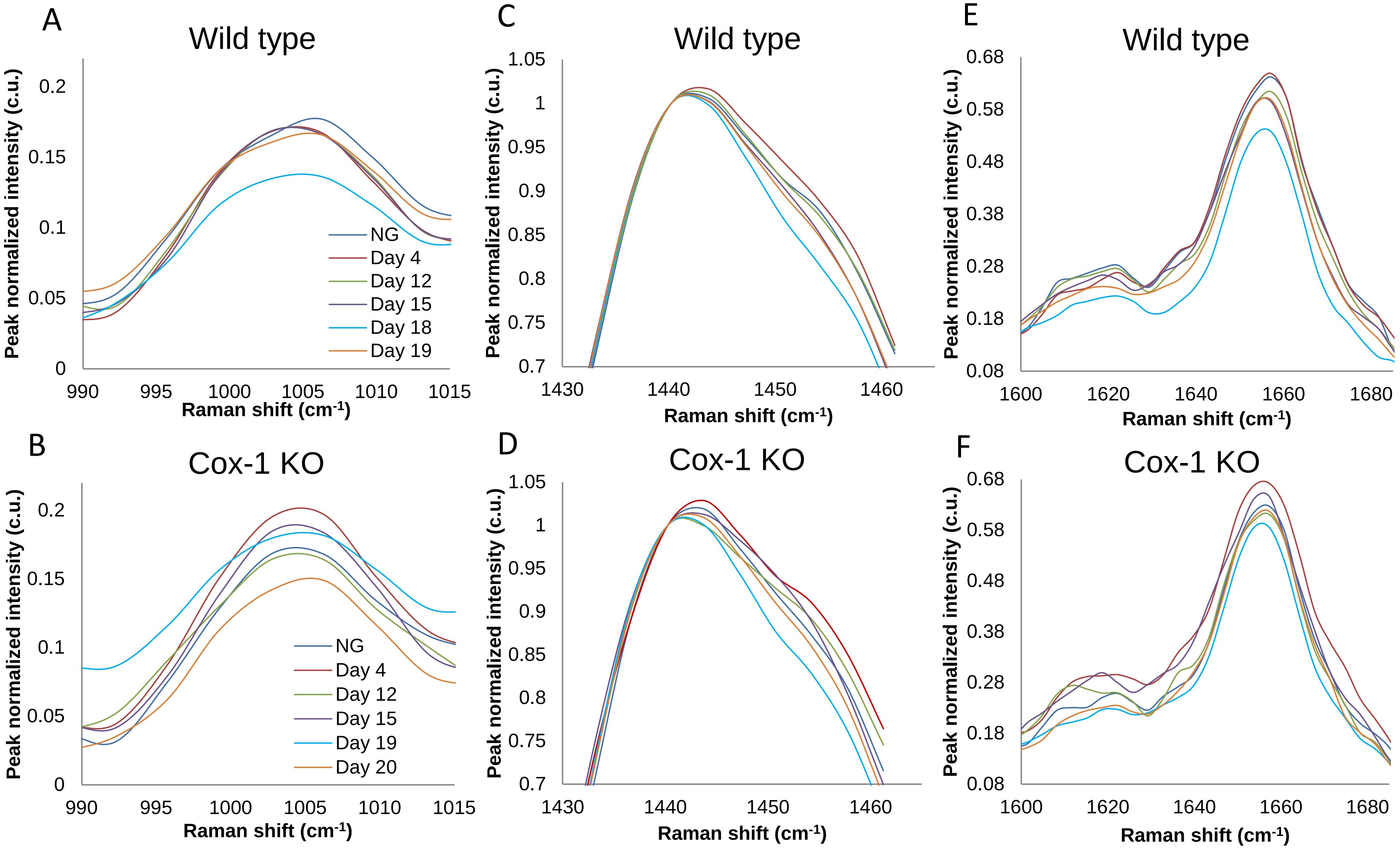

Supplemental Figure 1. Average Raman spectra from the 1003 cm^{-1} peak (A & B), 1440 cm^{-1} peak (C & D), and 1657 cm^{-1} peak (E & F) during different pregnancy time points in WT and Cox-1 KO mice. Spectra were peak-normalized to the 1440 cm^{-1} peak for ease of visualization.

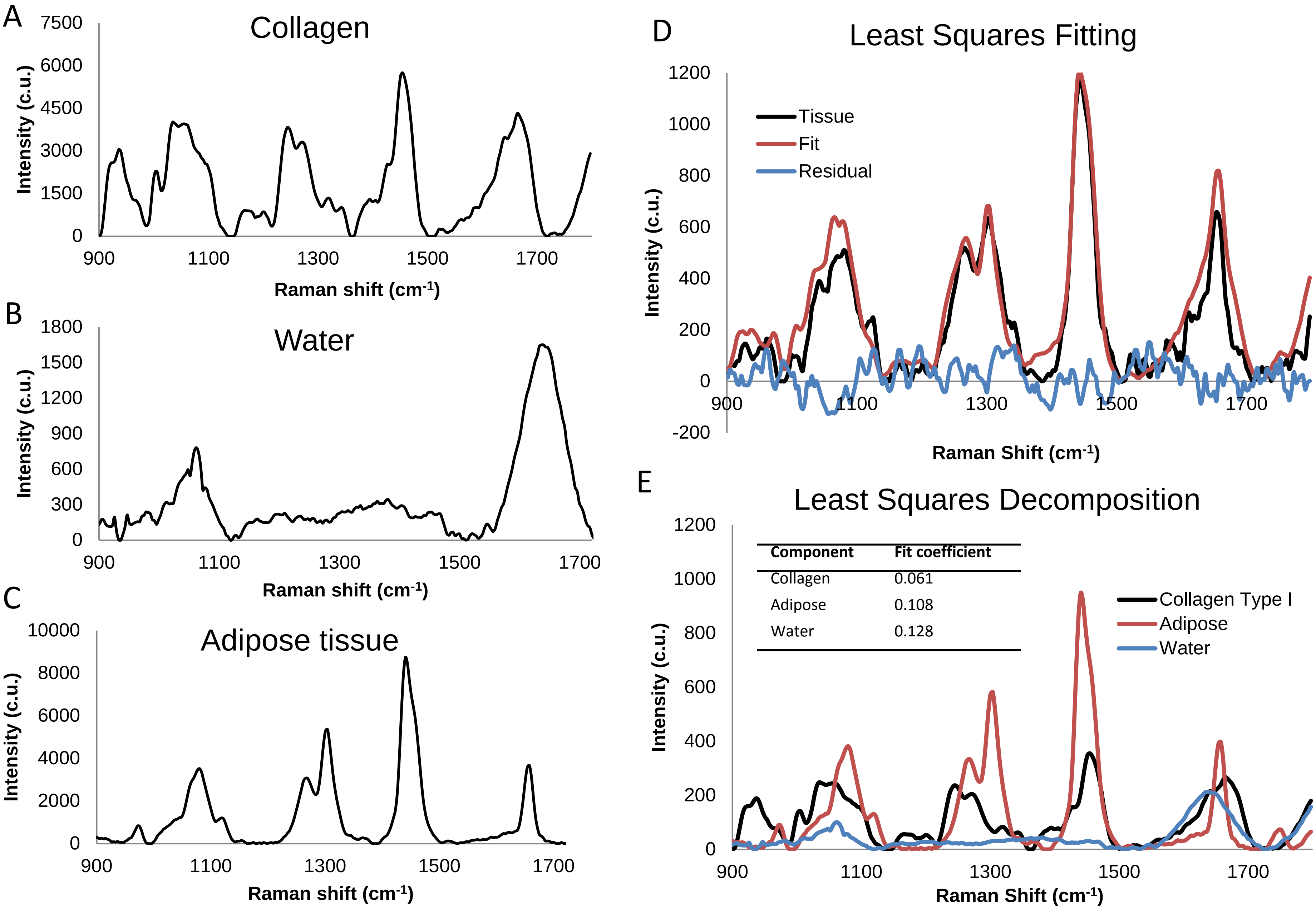

Supplemental Figure 2. Non-negative least squares components and model fits. Raman spectra of A) collagen I, B) water, and C) mouse adipose tissue. D) Overlay of representative cervical tissue spectrum (black), least squares fit (red), and the residual (blue). E) Pure spectra multiplied by their fit coefficients from the tissue spectrum in D, with their fit coefficients inlaid in the plot.

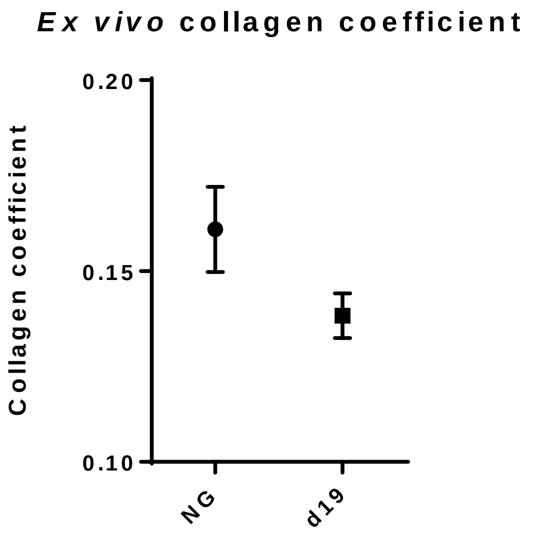

Supplemental Figure 3. Non-negative least squares collagen coefficients obtained from excised non-gravid (n=8) and day 19 (n=4) WT cervix tissues. Plotted as mean ± SEM, ($p=0.05$).
